# Central Nervous System Involvement in Splenic Marginal Zone Lymphoma

**DOI:** 10.7759/cureus.81250

**Published:** 2025-03-26

**Authors:** Kabeer Ali, Jennifer Miatech, Falguni Patel, Abhinav Karan, Walter JR Quan

**Affiliations:** 1 Internal Medicine, University of Florida College of Medicine – Jacksonville, Jacksonville, USA; 2 Hematology and Oncology, University of Florida College of Medicine – Jacksonville, Jacksonville, USA

**Keywords:** cns lymphoma, generalized seizure, intrathecal infusion, intrathecal therapy, marginal zone lymphoma mzl, marginal zone lymphoma (mzl), rituximab therapy, splenic marginal zone lymphoma, ventriculo-peritoneal shunt surgery, ventriculoperitoneal (vp) shunt

## Abstract

Marginal zone B-cell lymphoma (MZL) represents a heterogeneous group of indolent non-Hodgkin lymphomas (NHL) originating from the marginal zone of B cells in lymphoid tissues. Typically, MZL is classified as nodal, extranodal, and MZL with splenic involvement. Central nervous system (CNS) involvement is rare, whether it presents as a primary dural lymphoma or as a consequence of secondary CNS involvement. CNS involvement of MZL presents with non-specific symptoms such as headaches, focal neurological deficits, cognitive impairment, and seizures in the setting of mass effect. It is essential to consider new CNS infiltration as a possibility in patients with hematological malignancies who exhibit new neurologic symptoms. Herein, we present a case of a patient with a ventriculoperitoneal shunt and recently diagnosed with splenic MZL who presented with status epilepticus and was subsequently diagnosed with secondary CNS involvement, highlighting its associated diagnostic and therapeutic challenges.

## Introduction

Marginal Zone B-cell Lymphoma (MZL) is a rare and indolent type of non-Hodgkin lymphoma (NHL) originating from B cells in the marginal zone of lymphoid tissues. It is classified into three main subtypes; extranodal (also known as mucosa-associated lymphoid tissue or MALT lymphoma), nodal, and splenic marginal zone lymphoma (SMZL). These constitute 5-17% of all NHL [1]. In the United States, the incidence rate for MZL was reported to be 5.7 per 1,000,000 person-years for nodal MZL and 12.3 per 1,000,000 person-years for extranodal MZL [2]. Epidemiological data suggest that the median age at presentation for MZL varies between 50 and 69 years, with a greater incidence noted in males than females [3]. SMZL makes up less than 1% of all NHL [4]. The most common extranodal sites are the gastrointestinal tract, ocular/adnexal, and salivary glands.

Conversely, central nervous system (CNS) involvement is exceptionally uncommon, whether it presents as a primary dural lymphoma or as a consequence of secondary CNS involvement, and has only been documented in case reports. Due to the rarity of this presentation, the incidence of CNS involvement remains to be determined. In the setting of mass effect, CNS involvement of MZL presents with non-specific symptoms such as headaches, focal neurological deficits, cognitive impairment, and seizures [5].

Herein, we present a case of a patient with splenic MZL who presented with status epilepticus and was subsequently diagnosed with secondary CNS involvement. Although MZL cases are typically characterized as slow-growing, the prognosis for individuals with CNS involvement remains uncertain mainly due to the absence of standardized treatments and the tendency for relapse despite prompt diagnosis and intervention [6]. This case highlights the diagnostic and therapeutic challenges associated with an infrequent presentation of MZL with CNS involvement.

## Case presentation

**Figure 1:**
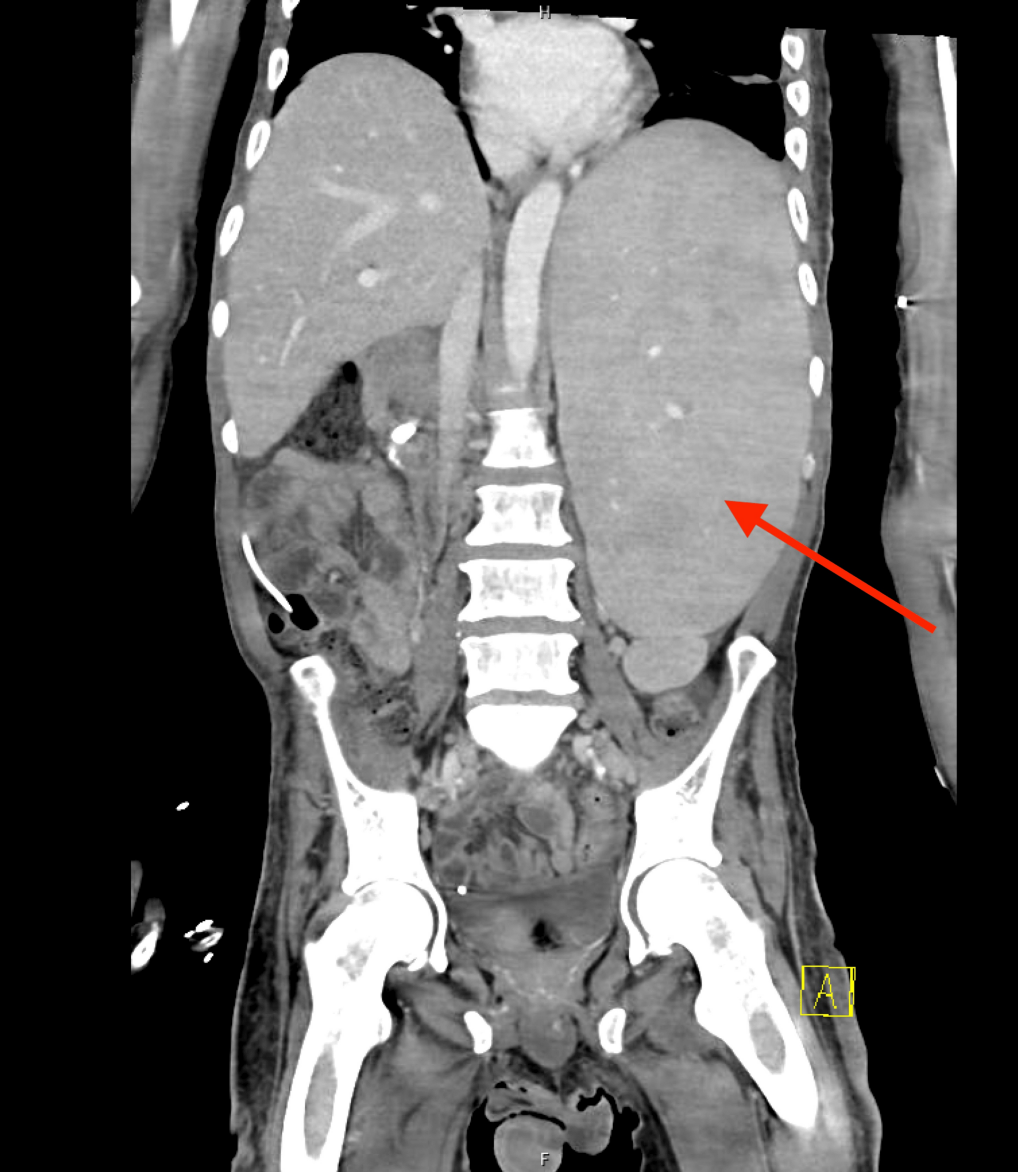
CT scan of the patient’s abdomen and pelvis demonstrating significant splenomegaly

**Figure 2:**
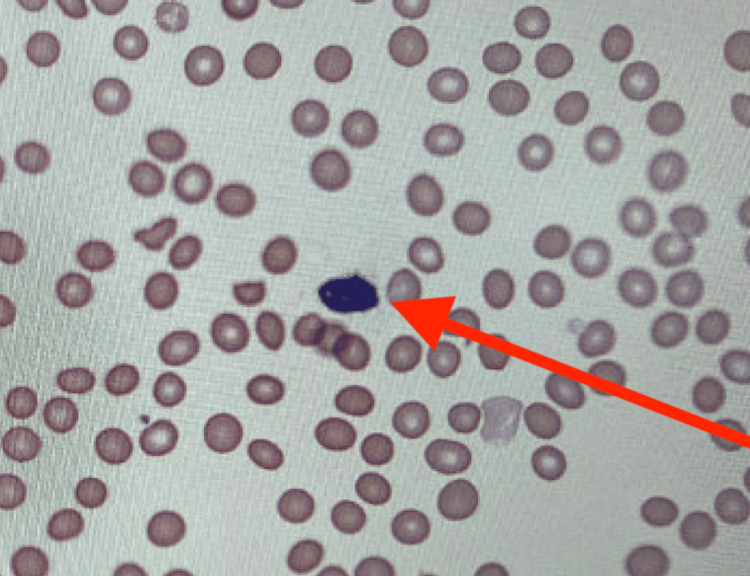
Peripheral blood smear highlighting a villous lymphocyte

A 49-year-old male with a past medical history of a seizure disorder and hydrocephalus with a ventriculoperitoneal shunt presented to the emergency department after a witnessed generalized tonic-clonic seizure (GTC). He was recently diagnosed with extranodal MZL with bone marrow involvement as an outpatient. His workup included a computed tomography (CT) scan of his abdomen and pelvis, which showed significant splenomegaly measuring 20.7 cm in craniocaudal dimension (Figure 1). Peripheral flow cytometry showed matured B-cell lymphoma involving 68 percent of live cells. Peripheral blood smear showed evidence of villous lymphocytes (Figure 2). His bone marrow evaluation confirmed mature B-cell lymphoma, which was CD20 positive, involving 70% of marrow cells. There was no overt increase in myeloblasts or plasma cells. Flow cytometry in the patient’s bone marrow revealed positivity in CD19, CD5, CD11c, CD23, and CD45. Cytogenetic and molecular studies showed a complex male karyotype 45-47 XY with deletions of 7q31 and TP53.

**Figure 3:**
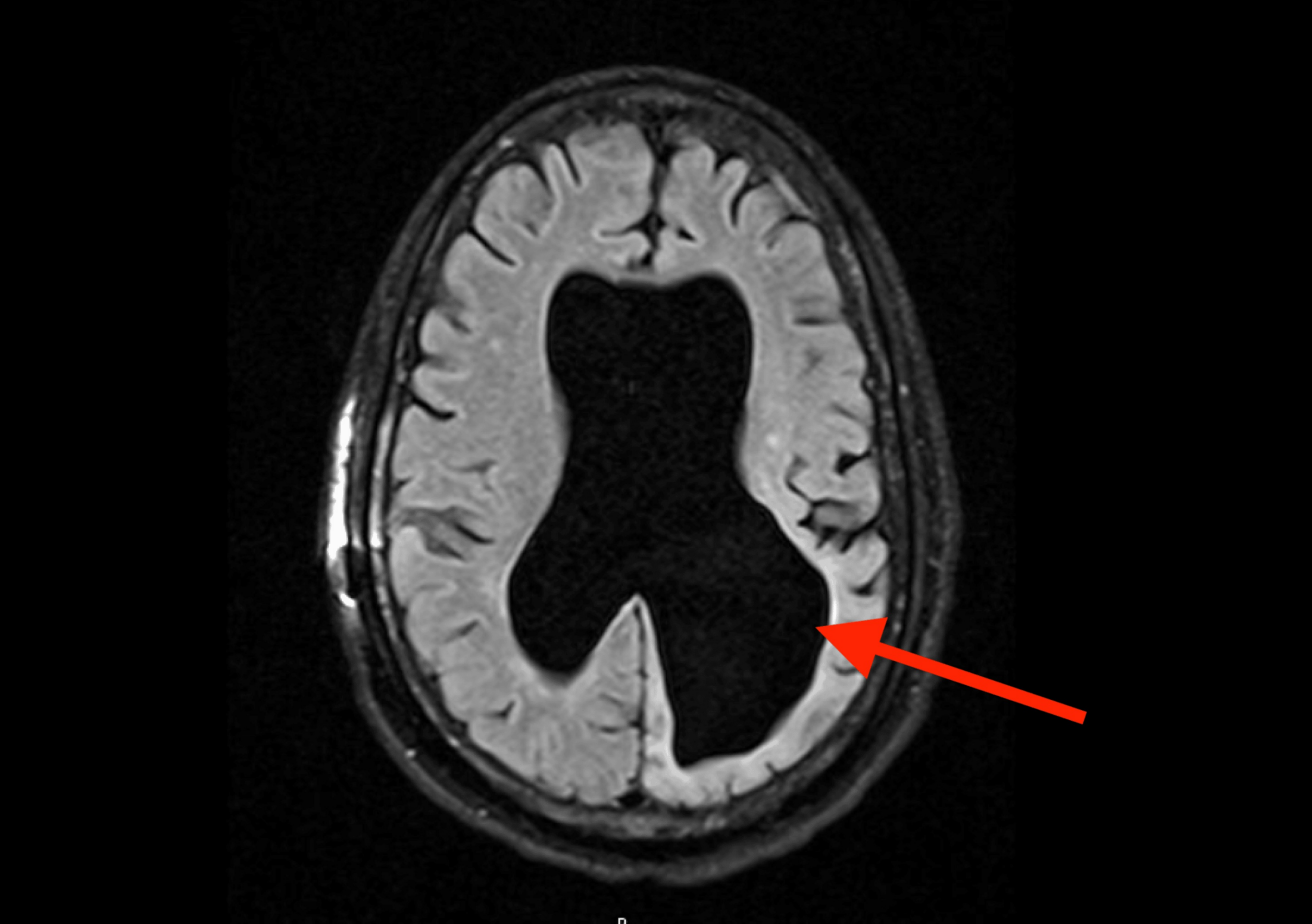
MRI brain showing stable biventricular dilatation and no evidence of leptomeningeal enhancement

Laboratory investigations showed a white blood cell count of 29 × 10^3^ cells per microliter (cells/µL) with an absolute lymphocytosis, hemoglobin of 6.9 g/dL, and a platelet count of 68 ×10^3^ cells/µL (Table 1). A magnetic resonance image (MRI) of the brain without contrast revealed no acute changes but stable chronic lateral ventricle dilatation and ventricular shunt placement (Figure 3). The patient developed GTC seizures refractory to intravenous levetiracetam. The patient was subsequently admitted to the neurocritical care unit (ICU) for status epilepticus. An electroencephalogram (EEG) confirmed seizure activity localized to the left hemisphere.

**Table 1:** Laboratory values at presentation with reference ranges for comparison

Laboratory Investigation	Patient Value	Reference Range
White blood cell	29 × 10^3^ cells/µL	4-11 × 10^3^ cells/µL
Hemoglobin	6.9 g/dL	11.6-15 g/dL
Platelet	68 × 10^3^ cells/µL	150-400 × 10^3^ cells/µL
Cerebrospinal fluid glucose	63 mg/dL	50-80 mg/dL
Cerebrospinal fluid protein	38 mg/dL	15-60 mg/dL

An infectious and toxicology workup was unremarkable, including hepatitis C and human herpesvirus-8 (HHV-8) testing, and electrolytes remained within normal limits. The ventricular shunt was evaluated by the neurosurgical team and was determined functional. A lumbar puncture revealed glucose of 63 mg/dL and protein of 38 mg/dL. Cytology was significant for rare lymphocytes. Flow cytometry of the cerebrospinal fluid (CSF) revealed 3% monoclonal B cells, concerning lymphoma involvement. The sample was clear, and no red blood cells were identified in the CSF sample, indicating no possible peripheral blood contamination. Due to the concern of primary CNS involvement of his MZL, he received one dose of intrathecal (IT) 12 mg methotrexate and 50 mg hydrocortisone. Subsequently, after IT treatment, the patient demonstrated no further seizure activity. The patient returned to baseline mentation and functional status. Repeat lumbar puncture after IT treatment revealed no further malignant cells. The patient was subsequently discharged with an outpatient treatment plan with Rituximab 375 mg/m2 weekly for four doses. Unfortunately, the patient was lost to follow-up.

## Discussion

MZL is a distinct low-grade lymphoma of the post-germinal center marginal zone B cells. The subtypes of MZL which share similar histopathologic and immunophenotypic features are extranodal or MALToma, nodal, and splenic MZBCL. MZL comprises only 5-17% of NHLs [4]. CNS involvement in MZL is uncommon and occurs in approximately 1-2% of cases. Despite its rarity, the incidence may be underestimated due to asymptomatic or unrecognized cases. Primary CNS involvement in MZL denotes the lymphoma originating within CNS structures, such as primary dural or intraocular lymphoma. Secondary CNS involvement, on the other hand, refers to MZL originating outside the CNS but demonstrating spread to CNS structures, such as dural or ocular regions. A small retrospective study evaluated 10 patients with MZL with CNS involvement. Six of these patients were found to have primary CNS involvement, and the remaining four had secondary CNS involvement [4]. A comprehensive literature review reveals that this condition is infrequently encountered, and most reported literature relates to the primary CNS involvement of MZL.

Patients with CNS involvement in MZL may present with a broad spectrum of neurological symptoms, including headaches, cognitive impairment, cranial nerve palsies, seizures, and focal neurological deficits. These manifestations often mimic other neurological conditions, necessitating a high index of suspicion for prompt diagnosis. Differential diagnoses of seizures in a patient with a ventriculoperitoneal shunt, such as shunt malfunction, infection, or metabolic disturbances, should be considered. According to a systematic review by Ayanambakkam et al., headaches are the most frequently reported symptom, followed by seizures [1]. In some cases, patients may be asymptomatic, and the diagnosis may be incidental, discovered during craniotomy for another indication [7]. Diagnosing CNS involvement in MZL relies on clinical assessment, neuroimaging studies, CSF analysis, and histopathological examination. Imaging findings may reveal leptomeningeal enhancement, parenchymal lesions, or both. CSF analysis may demonstrate lymphocytic pleocytosis, elevated protein levels, and the presence of atypical lymphoid cells, as was seen in our case. Some reported cases of secondary CNS lymphoma included non-specific imaging findings such as leptomeningeal, dural, subependymal, and cranial nerve enhancement [8]. In our case, the diagnosis was supported by monoclonal B cells within the CSF. In a retrospective study examining various cases of primary CNS lymphoma, tumors were predominantly situated in the frontal lobe.

Conversely, there needs to be corresponding data regarding secondary CNS MZL [9]. Cerebrospinal final analysis can aid the diagnosis as it acts as a reservoir of lymphomatous cells [10]. Most often, lymphomas demonstrate hematogenous spread to the CNS. There has been reported literature on low-grade B-cell CNS lymphoma manifesting as monoclonal B cells being detected in the CSF, similar to our case [11]. However, limited data is available regarding secondary CNS MZL, particularly concerning the efficacy of CSF analysis for its detection. It is established that in primary CNS lymphoma cases, CSF evaluation can yield negative results for lymphomatous cells. Several retrospective studies and case reports corroborate this finding [12]. Notably, a retrospective study on CNS EMZL patients revealed that none of those evaluated through cytological CSF analysis tested positive for malignancy [9]. MZL typically exhibits an indolent disease course, although it may behave more aggressively in rare instances.

Treatment options for SMZL with secondary CNS involvement lack guidance and are multifaceted and require a combination of systemic and CNS-directed therapies. Intrathecal therapy is usually achieved with methotrexate and cytarabine [13]. Acalabrutinib has shown efficacy in treating relapsed/refractory MZL, but its use in cases with secondary CNS involvement lacks specific clinical data [14]. For fit patients, high-dose chemotherapy followed by autologous stem cell transplantation (ASCT) can be considered. This case highlights an uncommon presentation of CNS involvement associated with MZL. Due to the rarity of CNS involvement, it is imperative to report such cases, as there are currently no standardized guidelines on diagnosis or treatment.

## Conclusions

This case demonstrates the diagnostic dilemma that occurs when faced with the exceedingly rare occurrence of involvement of the CNS in a patient with SMZL, especially in those with pre-existing neurologic pathology such as a ventriculoperitoneal shunt. This emphasizes the need for early CNS evaluation in patients with SMZL presenting with neurological symptoms, even in the absence of overt imaging findings. CNS involvement suggests an aggressive disease course. Further research is needed to understand better the mechanisms driving CNS dissemination in SMZL and to develop more effective treatment strategies for affected patients.
